# Non-coding RNAs in the pathophysiology of heart failure with preserved ejection fraction

**DOI:** 10.3389/fcvm.2023.1300375

**Published:** 2024-01-08

**Authors:** Elisabeth A. Jalink, Amber W. Schonk, Reinier A. Boon, Rio P. Juni

**Affiliations:** ^1^ Department of Physiology, Amsterdam University Medical Centers, Vrije Universiteit Amsterdam, Amsterdam, Netherlands; ^2^ Amsterdam Cardiovascular Sciences, Microcirculation, Amsterdam, Netherlands; ^3^ Institute for Cardiovascular Regeneration, Centre for Molecular Medicine, Goethe University Frankfurt am Main, Frankfurt am Main, Germany; ^4^ German Centre for Cardiovascular Research, Partner Site Frankfurt Rhein/Main, Frankfurt, Germany

**Keywords:** HFpEF, HFpEF pathophysiology, non-coding RNAs: miRNAs, lncRNAs, circRNAs, biomarkers, therapeutic targets

## Abstract

Heart failure with preserved ejection fraction (HFpEF) is the largest unmet clinical need in cardiovascular medicine. Despite decades of research, the treatment option for HFpEF is still limited, indicating our ongoing incomplete understanding on the underlying molecular mechanisms. Non-coding RNAs, comprising of microRNAs (miRNAs), long non-coding RNAs (lncRNAs) and circular RNAs (circRNAs), are non-protein coding RNA transcripts, which are implicated in various cardiovascular diseases. However, their role in the pathogenesis of HFpEF is unknown. Here, we discuss the role of miRNAs, lncRNAs and circRNAs that are involved in the pathophysiology of HFpEF, namely microvascular dysfunction, inflammation, diastolic dysfunction and cardiac fibrosis. We interrogated clinical evidence and dissected the molecular mechanisms of the ncRNAs by looking at the relevant *in vivo* and *in vitro* models that mimic the co-morbidities in patients with HFpEF. Finally, we discuss the potential of ncRNAs as biomarkers and potential novel therapeutic targets for future HFpEF treatment.

## Introduction

Heart Failure with preserved Ejection Fraction (HFpEF), defined as heart failure (HF) with a left ventricular ejection fraction (LVEF) of ≥50%, represents a single largest unmet clinical need in cardiovascular medicine given the high prevalence and health care burden, and limited effective treatments. In comparison to its counterpart HF with reduced EF (HFrEF), patients with HFpEF are generally older, display a higher proportion of females and higher prevalence of comorbidities, including diabetes, obesity, hypertension, chronic kidney disease, and atrial fibrillation, with less likelihood to have a myocardial infarction (1). In addition to the symptoms and signs of volume overload and a preserved EF, the diagnostic criteria of HFpEF include evidence of (1) structural LV remodeling as assessed by left atrial (LA) volume index or LV mass; (2) diastolic LV dysfunction assessed by early diastolic mitral inflow velocity (E), early diastolic mitral annular tissue velocity (e’), and their ratio (E/e’); (3) pulmonary hypertension indicated by peak tricuspid regurgitation velocity; and (4) increased myocardial wall stress indicated by increased plasma natriuretic peptide levels (2). Currently, either H_2_FPEF or HFA-PEFF scoring systems provides a reliable diagnostic algorithm to estimate the probability of the occurrence of HFpEF in patients that is applicable in clinical practice and trials settings (3, 4).

HFpEF constitutes a substantial portion of HF cases, ranging from 50% to 70% of all HF patients (5, 6), and currently affects 9% of people older than 60 years, with >6 million patients suffer from HFpEF in the US and EU combined (7). While there is a trend towards decreasing incidence of HFrEF (8), the incidence of HFpEF increases over time due to aging populations and rising co-morbidities, contributing to a substantial public health challenge (1). HFpEF incidence varies from 50% to 60% depending on the study cohorts, ranging from 250,000 to 300,000 cases annually (9, 10).

Patients with HFpEF face significant morbidity and mortality, with one-year mortality rates range from 10% to 30% (11), while five-year mortality rates raises to approximately 75% (12). Unfortunately, there is limited evidence-based effective treatment for HFpEF, which is attributed to the ongoing lack of understanding of HFpEF underlying mechanisms. The treatment for HFrEF has been proven inefficient to improve primary outcome in HFpEF patients, indicating different underlying pathophysiology. It is then imperative to further our understanding on the pathomechanisms of HFpEF to elucidate pathways or genes that can potentially be used as novel therapeutic targets.

## Pathomechanisms of HFpEF: current understanding

Compared to HFrEF, HFpEF patients are older and exhibit a higher burden of non-cardiac comorbidities (13), with the most prevalent ones being DM, obesity, hypertension, and renal dysfunction (14). These comorbidities contribute to cardiac remodeling through systemic inflammation and microvascular damage (15, 16). Pro-inflammatory state in HFpEF is indicative from the higher plasma levels of several inflammatory markers, including TNFα, IL1β, IL6 and C-reactive protein (17, 18). This chronic inflammation promotes damage on endothelium as the frontline of the vasculature, leading to systemic endothelial dysfunction, which is prevalent in HFpEF patients (19, 20) and underlining the potential of microvascular dysfunction as a therapeutic target in HFpEF.

Impaired cardiac microvascular function contributes to reduced coronary perfusion, promoting development of diastolic dysfunction (21). It also reflects dysfunction of cardiac microvascular endothelial cells (CMECs) and its paracrine signaling to cardiomyocytes. CMECs exhibit direct regulatory function on cardiomyocyte relaxation (22, 23). Inflammatory insults, such as TNFα and IL1β (23), and uremic sera from patients with renal insufficiency (22), impaired the endothelial-enhancement of cardiomyocyte relaxation. Improving endothelial function with SGLT2 inhibitor led to improvement of diastolic function (22, 23). Nitric oxide (NO) plays an important role to mediate the regulatory effect of endothelium. Nevertheless, endothelial-cardiomyocyte cross-talk goes beyond NO, as various other endothelial-derived molecules contribute to this cellular interaction (24, 25). Endothelial dysfunction leads to the imbalance between reduced availability of protective molecules and increased secretion of detrimental factors. Addressing endothelial dysfunction in HFpEF requires a comprehensive approach aiming at restoring equilibrium among these factors, and should not be limited only to targeting of a single endothelial-derived factor.

Cardiomyocyte remodeling in HFpEF involves cardiomyocyte hypertrophy, altered calcium handling, changes in myofilament properties, and imbalance myocardial energetics, which contribute to impaired diastolic function (5). Cardiomyocyte hypertrophy is one of the most common structural abnormalities associated with HFpEF. Ventricular myocardium from patients displays increased resting or diastolic tension due to an increase in actin-myosin cross-bridge activation as a result of elevated diastolic cytosolic calcium concentration, which is due to reduced sarcolemmal calcium extrusion due to sodium-calcium exchanger abnormalities (26). At the cellular level, isolated cardiomyocytes from HFpEF patients showed increased resting tension or passive stiffness (27), which is also dependent on titin, a large sarcomeric protein that functions as a molecular spring. The stiffness of titin is dependent on expression and phosphorylation of its compliant N2BA and stiff N2B isoforms (28–30). Titin phosphorylation is mediated by multiple enzymes, including PKA, PKC, CAMKII, as well as ERK-2, which is regulated by endothelin-1, and PKG, which is activated by NO (31), underlining multiple links of the contribution of endothelial cells on cardiomyocyte diastolic function.

Diastole is an active process that utilizes ATP. Increased energy consumption is associated with elevated diastolic tension in HFpEF (26). Myocardial phosphocreatine/ATP ratio was shown to be lower in patients with HFpEF as compared to control and was associated with diastolic dysfunction (32). These can be worsened by the presence of microvascular dysfunction and increased myocardial extracellular matrix (ECM) deposition, which increases the oxygen diffusion distance between the capillary and cardiomyocytes (33). Excessive deposition of ECM proteins leads to cardiac fibrosis, contributing to diastolic stiffness and impaired relaxation in HFpEF patients (34). The accumulation of ECM is preceded by the formation of myofibroblasts through activation of resident cardiac fibroblasts or mesenchymal transition of other cell types, including epicardial and endothelial cells. In addition, ECM synthesis can occur in the absence of myofibroblasts in hyperglycemic condition, which increases collagen production directly from fibroblasts (35). Several molecular drivers of the fibrotic process have been shown upregulated in HFpEF, including TGFβ, a potent inducer of fibroblasts-myofibroblasts switch, IL11 and Galectin-3. In addition, there were altered plasma levels of biomarkers that reflect collagen degradation, such as lower matrix metalloproteinase (MMP) and higher tissue inhibitor metalloproteinase (TIMP) (36), further underlining the involvement of fibrotic pathway dysregulation in HFpEF pathophysiology.

**Figure 1:**
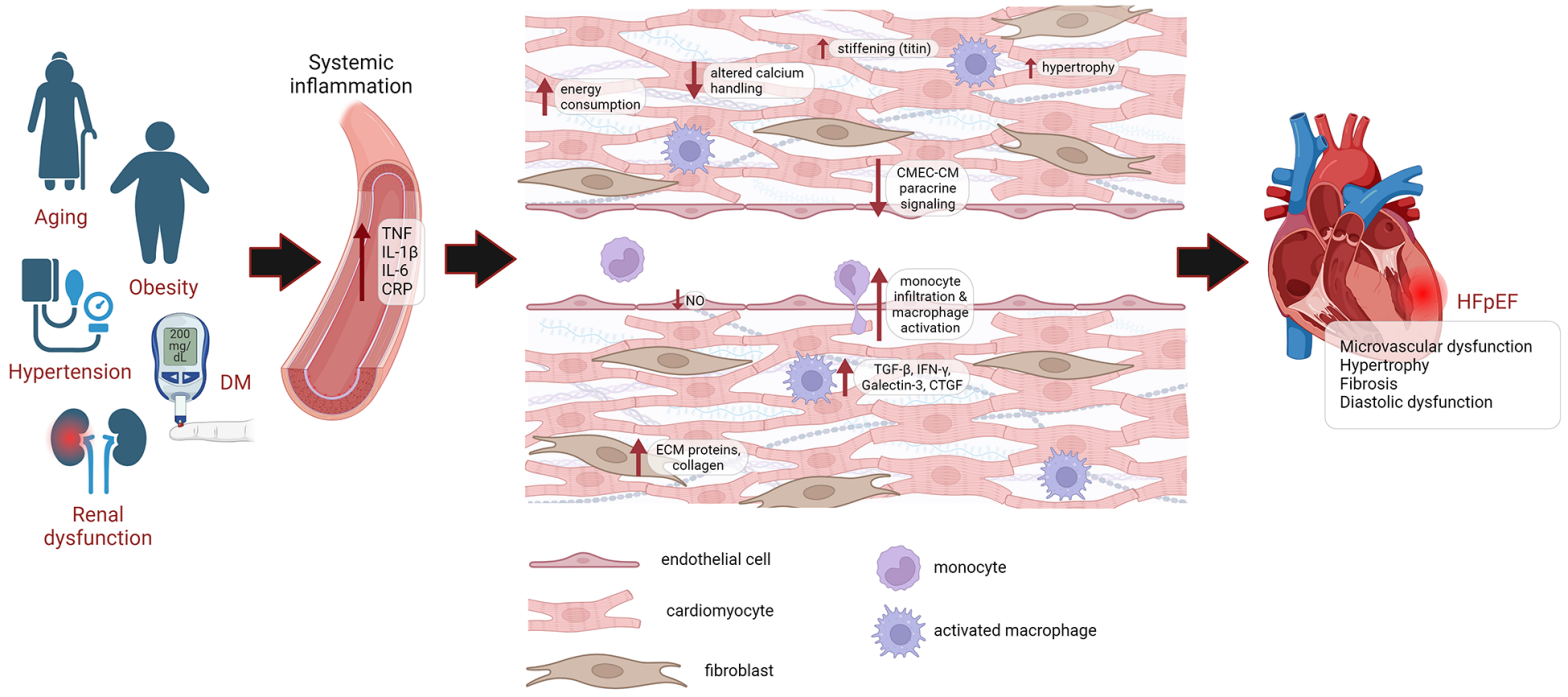
Pathophysiology of HFpEF. HFpEF patients are older, mostly female, and exhibit a higher burden of non-cardiac comorbidities, such as DM, obesity, hypertension and renal dysfunction. These comorbidities lead to systemic inflammation, causing microvascular damage. This drives endothelial dysfunction, leading to reduced NO levels, higher infiltration of monocytes and activation of cardiac resident macrophages, which express profibrotic factors TGFβ, IFNγ, Galectin-3 and CTGF, leading to proliferation and activation of cardiac fibroblasts, promoting the deposition of ECM proteins and collagen. Endothelial dysfunction also impairs paracrine signaling of CMECs to cardiomyocytes. Cardiomyocyte remodeling is also evident from cardiomyocyte hypertrophy, altered calcium handling, increased energy consumption and increased passive stiffness. Eventually, this leads to diastolic dysfunction and HFpEF. DM, diabetes mellitus; CRP, C-reactive protein; CMEC, cardiac microvascular endothelial cell; CM, cardiomyocyte; ECM, extracellular matrix; NO, nitric oxide; HFpEF, heart failure with preserved ejection fraction.

Collagen deposition in the HFpEF heart is associated with microvascular inflammation, which permits higher infiltration of monocytes and activation of cardiac resident macrophages due to reduced NO levels. These inflammatory cells express profibrotic factors, such as TGFβ, IFNγ, Galectin-3 and CTGF, which induce proliferation and activation of cardiac fibroblasts into myofibroblasts, promoting ECM deposition (35, 37) (Figure 1).

## Non-coding RNAs in pathophysiological processes leading to HFpEF

With the advancement of the sequencing technologies, it is revealed that ∼98% of our genome is non-coding, and merely −2% are coding for proteins. These non-coding RNAs (ncRNAs) function as epigenetic regulators of gene expression and are involved in biological processes through various distinct molecular mechanisms. The abundance of ncRNAs in the cardiovascular system and the aberrant expression of ncRNAs in cardiac development and cardiac diseases indicate their significance in cardiovascular physiology and pathology.

Extensive research has highlighted the significant involvement of ncRNA in the development of cardiac diseases. However, our knowledge on their contribution to the evolution of HFpEF is still at its infancy. This is partly due to the lack of animal models that can sufficiently recapitulate human HFpEF. Preclinical modeling of HFpEF should ideally comply with a preserved EF of ≥50%, diastolic dysfunction, exercise intolerance, pulmonary edema and concentric cardiac hypertrophy, the characteristics compatible to the patients (38). Several pre-clinical models have been used to study HFpEF pathomechanisms. Single hit *in vivo* models, which integrate only single co-morbidity, display some typical signs of HFpEF, and therefore may approximate subsets of HFpEF patients. Several single hit models used to study HFpEF are: (1) hypertension model induced by Angiotensin-II (Ang-II) infusion or genetic sensitivity to salt (39–46), (2) aging model, induced by natural aging or genetically accelerated (senescence accelerated mouse, SAM) (47–51), (3) obesity or diabetes, induced by high fat or western type diet, leptin receptor (*db/db*) or leptin (*ob*/*ob*) deficiency (52–61). Most of the single hit models comply to some extent with H_2_FPEF or HFA-PEFF scoring systems. In some cases systolic dysfunction and reduced LVEF still develops, or other clinical signs are lacking, such as decreased exercise tolerance (62). Most multi hit models are based on obesity induced by high fat diet (HFD) in combination with other stressors, including hypertension (L-NAME, Ang-II, DOCP), both aging and Ang-II, or both aging and DOCP, to induce the HFpEF phenotype (63–66). Multi hit animal models better resemble the human HFpEF and should be advocated for future research in pre-clinical HFpEF study, as HFpEF is a multifactorial disease with diverse phenotypes.

**Figure 2:**
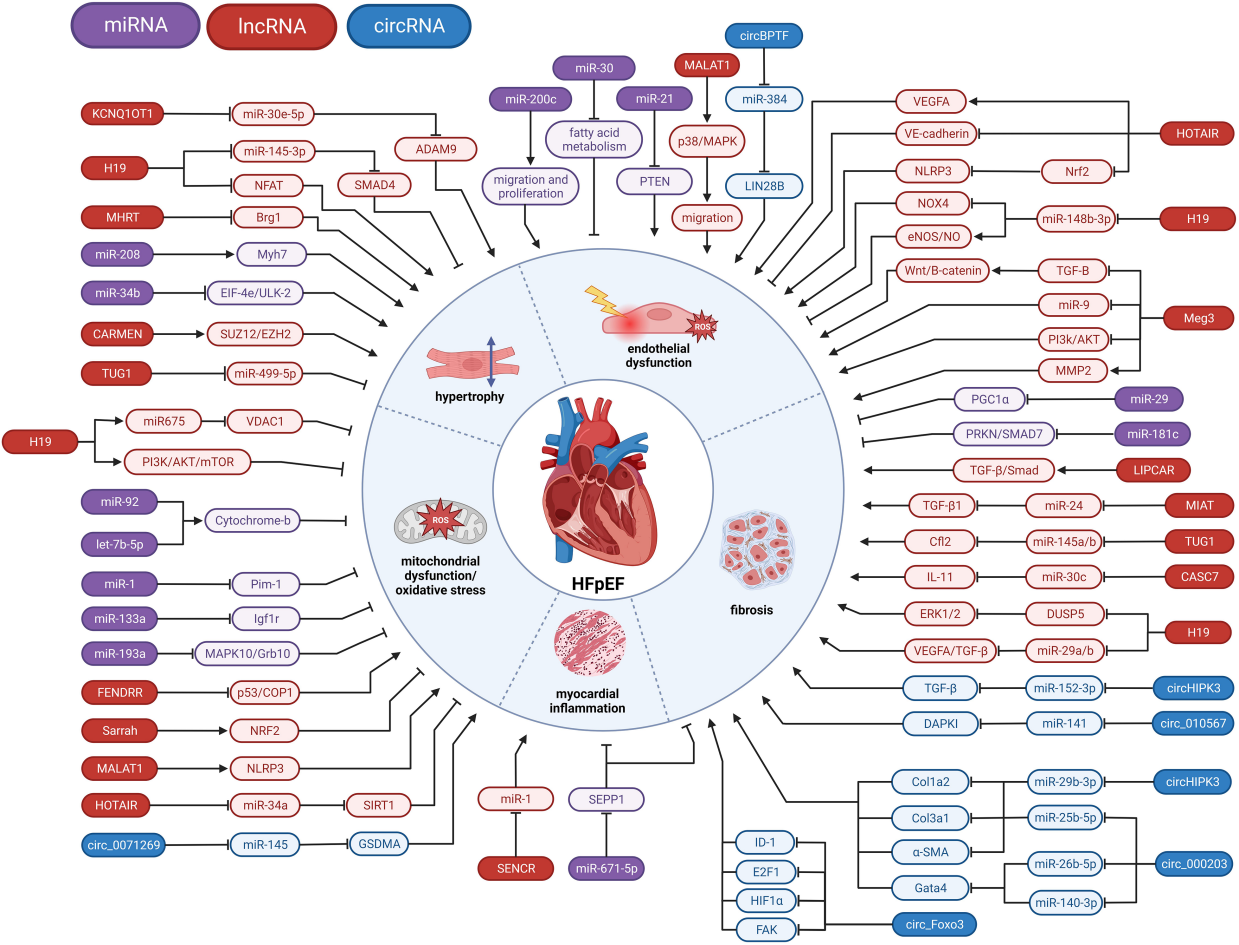
Involvement of ncRNAs in HFpEF pathophysiology. miRNA, microRNA; lncRNA, long non-coding RNA; circRNA, circular RNA; ⊣, inhibition; →, activation.

In this review, we gather the current knowledge of the role of ncRNA, including microRNAs (miRNAs), long noncoding RNAs (lncRNAs), and circular RNAs (circRNAs) in several pathophysiological pathways associated with HFpEF, such as microvascular inflammation, cardiac hypertrophy, diastolic dysfunction and interstitial fibrosis (summarized in Figure 2 and Table 1). We look at the clinical as well as subsets of relevant pre-clinical models, e.g., diabetic, obese or systemic hypertensive, to understand the role of ncRNA that may play a role in HFpEF pathophysiology. Harnessing this knowledge will provide novel insights into HFpEF pathomechanisms and offer a novel and innovative class of therapeutic targets for HFpEF.

**Table 1:** Specific function of ncRNAs in HFpEF pathophysiology.

Type of ncRNA	ncRNA	Dysregulation of the ncRNA in HFpEF	Target	Specific pathologies induced by the ncRNA in HFpEF	Reference
miRNA	miR-1	Up	Pim-1	Mitochondrial dysfunction/oxidative stress in EC/CM/CF	(67)
	miR-21	Down/up	PTEN	Endothelial dysfunction	(68)
	miR-29	Down	PGC1α	Fibrosis	(69)
	miR-30	Down/up	Fatty acid metabolism	Endothelial dysfunction	(70)
	miR-34	Down	EIF-4e/ULK-2	Hypertrophy	(71)
	miR-92	Down/up	Cytochrome-b	Mitochondrial dysfunction/oxidative stress	(72)
	miR-133a	Up	Igf1r	Mitochondrial dysfunction/oxidative stress	(73)
	miR-181	Up	PRKN/SMAD7	Fibrosis	(74)
	miR-193	Up	MAPK10/Grb10	Mitochondrial dysfunction/oxidative stress	(73)
	miR-200c	Up	Migration and proliferation	Endothelial dysfunction	(75)
	miR-208	Up	Myh7	Hypertrophy	(76)
	miR-671-5p	Up	SEPP1	Myocardial inflammation	(77)
	let7	Down	Cytochrome-b	Mitochondrial dysfunction/oxidative stress	(72)
lncRNA	FENDRR	Up	p53/COP1	Mitochondrial dysfunction/oxidative stress	(78)
	CARMEN	Up	SUZ12/EZH2	Hypertrophy	(79)
	MHRT	Up	Brg1	Hypertrophy	(80)
	SENCR	Up	miR-1	Myocardial inflammation	(81)
	LIPCAR	Up	TGF-β/Smad	Fibrosis	(82)
	MIAT	Up	miR-24/TGF-β1	Fibrosis	(83)
	CASC7	Up	miR-30c/IL-11	Fibrosis	(84, 85)
	TUG1	Down/up	miR-145a/b/Cfl2	Fibrosis	(86)
			miR-499-5p	Hypertrophy	(87)
	MALAT1	Down	p38/MAPK/migration	Endothelial dysfunction	(88)
			NLRP3	Mitochondrial dysfunction/oxidative stress	(89)
	Meg3	Up	MMP2	Fibrosis	(90)
			PI3k/AKT	Endothelial dysfunction	(91)
			miR-9	Endothelial dysfunction	(92)
			TGF-β/Wnt/B-catenin	Endothelial dysfunction	(92, 93)
	HOTAIR	Down/up	Nrf2/NLRP3	Endothelial dysfunction	(94)
			VE-cadherin	Endothelial dysfunction	(95)
			VEGFA	Endothelial dysfunction	(95)
			miR-34a/SIRT1	Mitochondrial dysfunction/oxidative stress	(96)
	KCNQ1OT1	Down	miR-30e-5p/ADAM9	Hypertrophy	(97)
	SARRAH	Down	NRF2	Mitochondrial dysfunction/oxidative stress	(98)
	H19	Up	miR-148b-3p/NOX4/eNOS/NO	Endothelial dysfunction	(99)
			miR-29a/b/VEGFA/TGF-β	Fibrosis	(100)
			DUSP5/ERK1/2	Fibrosis	(101)
			miR-675/VDAC1	Mitochondrial dysfunction/oxidative stress	(102, 103)
			PI3K/AKT/mTOR	Mitochondrial dysfunction/oxidative stress	(104)
			NFAT	Hypertrophy	(105)
			miR-145-3p/SMAD4	Hypertrophy	(103)
circRNA	circRNA_010567	Up	miR-141/DAPKI	Fibrosis	(106)
	circRNA_000203	Up	miR-26b-5p/Col1a2/3a1/α-SMA	Fibrosis	(107)
			miR-26b-5p/Gata4	Fibrosis	(108, 109)
			miR-140-3p/Gata4	Fibrosis	(108, 109)
	circFoxo3	Up	ID-1	Fibrosis	(110, 111)
			E2F1	Fibrosis	(110, 111)
			HIF1α	Fibrosis	(110, 111)
			FAK	Fibrosis	(110, 111)
	circHIPK3	Down	miR-29b-3p/Col1a2/3a1/α-SMA	Fibrosis	(112, 113)
			miR-152-3p/TFG-β	Fibrosis	(114)
	circBPTF	Up	miR-384/LIN28B	Endothelial dysfunction	(115)
	circ_0071269	Up	miR-145/GSDMA	Mitochondrial dysfunction/oxidative stress	(116, 117)

miRNA, microRNA; lncRNA, long non-coding RNA; circRNA, circular RNA.

### miRNAs in the pathophysiology of HFpEF

MicroRNAs (miRNAs) are small, single stranded, non-coding RNA molecules of around 22–25 nucleotides long (118, 119). miRNA biogenesis can proceed via the canonical or the non-canonical pathway, where it is not processed by the RNase III endonuclease Dicer in the cytoplasm (118, 119). The mammalian genome codes for more than 2000 miRNAs, and around 60% of coding genes are regulated by miRNAs (120). They regulate translational repression or mRNA degradation by binding to mRNAs in the miRNA response elements (MREs), usually located in the 3’untranslated region (UTR). One miRNA can target multiple target mRNAs and one mRNA can be targeted by multiple miRNAs (118).

#### miR-1

In a mouse model of type-1 diabetes mellitus (DM) induced-cardiomyopathy induced by streptozotocin (STZ), the mice displayed signs of diastolic dysfunction with preserved ejection fraction, and miR-1 expression was upregulated in the left ventricle (LV) and intensified as the diabetes progression continued. Pim-1 was shown to be a target of miR-1 and overexpression of Pim-1 alleviated diastolic dysfunction (67). In an *in vitro* model of rat cardiomyocytes and murine cardiac progenitor cells subjected to high glucose, Pim-1 expression was reduced. miR-1 inhibition led to the restoration of Pim-1 expression and the activation of the upstream regulator Akt. This resulted in an increase of survival signaling through upregulation of pBad and Bcl-2 expression and reduction of caspase activity.

Interestingly, in rat aortic banding model of HFrEF with predominant systolic dysfunction, downregulation of miR-1 was observed (121). miR-1 overexpression reduced cardiac dysfunction and the expression of hypertrophic gene markers and restored the expression and activity of calcium homeostasis genes, reduced fibrosis and pro-fibrotic genes and decreased apoptosis. These contradictory findings of miR-1 regulation could possibly be explained by the different disease models used, reflecting the different pathomechanisms between HFpEF and HFrEF.

#### miR-21

Circulating miR-21 levels were upregulated in old, frail, type-2 diabetes mellitus HFpEF patients, compared to age-matched healthy controls, and was reduced after 3-month treatment with SGLT-2 inhibitor empagliflozin (122). Elevated levels of circulating miR-21 were also observed in obese patients, which exhibited a correlation with diastolic dysfunction and concomitant with increased plasma levels of TGF-β and Smad3 and decreased Smad7. miR-21 upregulation was also linked to increased fibrosis markers, including elevated mRNA plasma levels of α-SMA, Collagen-I and Collagen-III (123). Interestingly, cardiac miR-21 levels were shown to be downregulated in type-2 DM db/db mouse model, leading to diastolic dysfunction with preserved ejection fraction (124). Decreased expression of miR-21 resulted in increased ROS formation, decreased NO bioavailability, and enhanced cardiac hypertrophy, leading to impaired cardiac diastolic function. At cellular level, exosomes derived from cardiac stromal cells from HF patients with reduced miR-21-5p levels reduced endothelial tubulogenesis, increased cardiomyocyte apoptosis and promotes transition of fibroblasts to myofibroblasts. The discrepancy between circulating and cardiac miR-21 levels may be due to miR-21 disposal out of cells upon injury, leading to increased plasma levels and reduced tissue expression. The therapeutic potential of miR-21 was shown as treatment with a miR-21 mimic decreased levels of PTEN, increased Akt activation and reduced levels of Caspase-3 and PCD4, leading to mitigation of cardiac dysfunction (68). In contrast, cardiac miR-21 increased in pressure overload mouse model induced by transverse aortic constriction (TAC) (125), which better resembles HFrEF. This is similar to miR-1 which is differently regulated in HFpEF vs. HFrEF model, again underlining different molecular pathways driving the development of these diseases.

#### miR-29

miR-29 is mostly known for its role in cardiac fibrosis and its plasma level changed in HF patients (126–128). miR-29 expression is downregulated in type-1 DM rat model with diastolic dysfunction and preserved ejection fraction (129). Deletion of miR-29 in mice led to the development of HFpEF, characterized by a fibrotic LV, diastolic dysfunction, pulmonary congestion, systemic hypertension and vascular remodeling (69). PGC1α, a driver of metabolic pathways in the cardiovascular system and important in mitochondrial biogenesis, was found to be the main target of miR-29, as it is upregulated in miR-29 KO mice, as well as in diabetic patients with dilated cardiomyopathy (DCM). Hypertension and HFpEF induced by miR-29 deficiency can be rescued by PGC1α haploinsufficiency, which reduces pathological cardiac mitochondrial accumulation and increases survival (69).

#### miR-30

miR-30b plasma levels were found downregulated in HFpEF patients (130, 131). In combination with 6 other miRNA plasma levels (let-7a-5p, miR-107, miR-125a-5p, miR139-5p, miR-150-5p and miR-342-3p), miR-30b-5p levels were able to discriminate between HFpEF and HFrEF patients, where miR-30b-5p levels were reduced in HFpEF compared to HFrEF (131), showing the potential of this miRNA as a biomarker of HFpEF. Plasma levels of miR-30c were also found to be lower expressed in HF, and in combination with BNP, miR-221, miR-328, and miR-375 plasma levels, was able to differentiate between HFpEF and HFrEF patients, where miR-30c levels tend to be lower in HFrEF compared to HFpEF (132).

miR-30 may drive HFpEF through its regulatory role on endothelial function. It was shown in db/db mouse and Goto-Kakizaki rat model for type-2 DM-associated diastolic dysfunction that miR-30d-5p and miR-30e-5p levels in circulating extracellular vesicles (EVs) were upregulated (70). Both miRNAs were also upregulated in the LV and microvascular endothelial cells *in vivo*, as well as in an *in vitro* culture of HUVECs upon senescence. Their overexpression in HUVECs induced oxidative stress and endothelial dysfunction, while the inhibition *in vivo* decreased oxidative stress and DNA damage in microvascular endothelial cells, potentially via the regulation of fatty acid metabolism, showing their potential as a therapeutic target for endothelial-driven pathogenesis of HFpEF (70).

#### miR-34

Lower levels of miR-34a were found in DM patients with LV diastolic dysfunction (LVDD) as compared to DM patients without LVDD, and in women with kidney dysfunction with LVDD as compared to women with kidney dysfunction without LVDD (133). Further, there was a positive association between plasma miR-34a levels in patients with LVDD with microvascular injury marker Angiopoietin-2. Interestingly, progression to HFpEF increased miR-34a as well as Angiopoietin-2 levels in women with DM (133). Similarly, in a rat model of HFD and STZ-induced diabetic cardiomyopathy with diastolic dysfunction, miR-34a-5p levels in the myocardium were elevated, along with increased collagen deposition, apoptosis and decreased Bcl-2 levels (134). Another study linked miR-34a expression to aging, the main risk factor for HFpEF, and a role in aging-induced apoptosis of cardiomyocytes via the inhibition of PNUTS protein levels (135). In addition, *in vitro* culture of H9c2 rat cardiomyocyte cell line under high glucose treatment showed increased apoptosis and miR-34a expression, and decreased expression of Bcl-2, the downstream target of miR-34a, whereas inhibition of miR-34a reduced apoptosis (134). In addition to miR-34a-5p, miR-34b-3p was shown to be decreased in ventricular heart biopsies of rats with sensory neuropathy-induced diastolic dysfunction, with EIF-4e and ULK-2 were found as the possible downstream targets (71). These studies show two related miRNAs, namely miR-34a-5p and miR-34a-3p that are differently regulated by aging and in 2 different HFpEF-related pre-clinical models, suggesting their specific regulation by different HFpEF-inducing factors.

#### miR-92

In old, frail, type-2 diabetes HFpEF patients, circulating miR-92 levels were upregulated compared to age-matched healthy controls, and the levels were reduced 3 months after treatment with SGLT-2 inhibitor empagliflozin (122). In a type-2 diabetic cardiomyopathy model of db/db mice, miR-92a-2-5p expression in cardiac mitochondria was reduced (72). Furthermore, cardiac ROS levels were increased due to decreased mitochondrial Cytochrome-b gene expression. Overexpression of miR-92a-2-5p in the db/db mice increased Cytochrome-b expression, reduced ROS production and lipid deposition, and improved cardiac diastolic dysfunction. Similarly, miR-92a-2-5p overexpression in neonatal rat ventricular cardiomyocytes (NRVMs) increased Cytochrome-b levels and decreased mitochondrial ROS levels and cardiomyocyte apoptosis (72).

#### miR-133a

Single-nucleotide polymorphisms (SNPs) in the gene for miR-133a are associated with impaired cardiac diastolic function in type-2 DM patients (136). In a C57BL/6J mouse model for type-1 DM-induced cardiomyopathy, miR-133a-3p expression was upregulated (73). Expression levels of miR-133a-3p were also upregulated in NRVMs under high glucose treatment. It was shown that the target for miR-133a-3p was Igf1r, a component of the IGF1R/PI3K/AKT signaling pathway, which is important for cell survival (73). In contrast, cardiac miR-133a was reduced in TAC mouse model and an *in vitro* fibrosis model using neonatal rat primary ventricular fibroblasts treated with Ang-II (137), again indicating that miRNAs can be differently regulated in diastolic vs. systolic HF.

#### miR-181

Circulating miR-181c levels were found to be upregulated in HFpEF patients that respond poorly to exercise training (138) and in patients diagnosed with HFpEF and DM, as compared to healthy age-matched controls (74). In line, miR-181a-2-3p was upregulated in the hearts of a rat model for diastolic dysfunction induced by sensory neuropathy (71). It was shown in adult human cardiac fibroblasts that miR-181c targets PRKN and SMAD7, indicating that miR-181 might be important in the development of fibrosis during HFpEF (74).

#### miR-193

In a mouse model for type-1 DM-induced cardiomyopathy, the expression of both miR-193a-3p and miR-193b-3p was upregulated (73). miR-193a-3p was also upregulated in NRVMs under high glucose treatment. miR-193a-3p binds to MAPK10 and Grb10, integral components of the IGF1R/PI3K/AKT signaling pathway important for cell survival. miR-193b has been also shown upregulated in obese ZSF-1 leptin-receptor knockout rat HFpEF model, particularly in the pulmonary arteries (PAs) and PA vascular smooth muscle cells (PAVSMCs) (139). In line, miR-193b expression was also heightened in the PAVSMCs from DM patients. miR-193b expression in rat PAVSMCs was increased by H_2_O_2_ treatment and directed towards the targeting of Nuclear factor Y α subunit (NFYA). The increased miR-193b expression is possibly due to ROS-dependent H3K9 acetylation, thereby enhancing NFYA transcript degradation and reducing NFYA expression, leading to reduced sGCβ1 promoter activation and transcription, an enzyme important for smooth muscle relaxation and vasodilation (139).

#### miR-200c

Circulating miR-200c was upregulated and positively correlated with diastolic dysfunction indices, LV mass and LV relative wall thickness in patients with psoriasis, which is characterized by chronic inflammation (140). miR-200c-3p levels were increased in EVs derived from primary NRVMs with induced hypertrophic phenotype (75). Furthermore, exposure to hypertrophic cardiomyocyte-derived EVs and direct overexpression of miR-200c-3p in HUVECs impaired endothelial angiogenic capacity. Silencing of miR-200c-3p in mice subjected to chronic pressure overload resulted in attenuated hypertrophy, a smaller fibrotic area, and higher capillary density. miR-200c-3p affects endothelial function by targeting genes that directly affect endothelial cell proliferation and migration (75), suggesting that miR-200c contributes to the regulation of cardiac diastolic function, possibly by targeting cardiac endothelial function.

#### miR-208

Circulating levels of miR-208a were upregulated in HFpEF patients as compared to healthy controls (141). In line, miR-208b was expressed higher in isolated peripheral blood mononuclear cells from HFpEF patients with hypertension compared to hypertension patients without HFpEF (142). The therapeutic potential of miR-208a was shown in Dahl salt-sensitive rat model for HF with diastolic dysfunction, where administration of antimiR-208a lead to cardiomyocyte hypertrophy and fibrosis, improved cardiac function, and increased survival (76).

#### miR-212

miR-212 was found upregulated in the LV tissue from patients with end-stage HF (143). In a rat model of chronic kidney disease (CKD)-induced HFpEF, miR-212 was overexpressed in the LV compared to healthy controls (144). Similarly, in rat model of radiation-induced diastolic dysfunction with preserved ejection fraction, cardiac miR-212 was upregulated (145). While FoxO3 was shown to be the target of miR-212 in HFrEF mouse model (146), it is not the case in HFpEF (144, 145), leading to poor understanding on molecular mechanism by which miR-212 influences hypertrophy in this model.

#### miR-671-5p

miR-671-5p was found to be a modulator of fibrosis (77). In an Ang-II infusion mouse model for diastolic dysfunction and LV hypertrophy, miR-671-5p was increased in fibroblasts, but not in endothelial cells or cardiomyocytes. Overexpression of miR-671-5p in HCFs activated fibrosis marker α-SMA and pro-inflammatory cytokines IL-6 and IL-8 via targeting Selenoprotein P1 (SEPP1). Antifibrotic treatment in Ang-II-induced diastolic dysfunction mouse model and in a Dahl salt-sensitive rat model for hypertension-induced diastolic dysfunction improved the diastolic function (77).

#### let-7

Plasma levels of let-7a-5p were downregulated in patients with chronic HF, and combined with the levels of 6 other miRNAs (miR-107, miR-125a-5p, miR139-5p, miR-150-5p, miR-30b-5p and miR-342-3p) can be utilized to discriminate HFpEF from HFrEF patients (131). Similarly, in a sensory neuropathy rat model for diastolic dysfunction, let-7a-5p was downregulated in the heart (71). Further, let-7b-5p was found to be downregulated in the mitochondria of a mouse diabetic cardiomyopathy model. Treatment of NRVMs with a let-7b-5p mimic increased Cytochrome-b expression, a negative regulator of mitochondrial ROS. Overexpression of let-7b-5p decreased mitochondrial ROS levels and apoptosis in cardiomyocytes (72). Contrary to let-7a and let-7b, let-7f-5p levels were upregulated in the hearts of mice with type-1 DM-induced cardiomyopathy (73). Furthermore, SNPs in the gene for let-7f were associated with impaired cardiac diastolic function in type-2 DM patients (136).

### lncRNAs in the pathophysiology of HFpEF

lncRNAs make up the largest and functionally most diverse group within the non-coding transcriptome (147). Much like protein-coding transcripts, many lncRNAs undergo post-transcriptional modifications, including alternative splicing, 5’-capping, and polyadenylation (119, 147). Additionally, some lncRNAs are formed through backsplicing events of linear mRNA, resulting in more stable circular RNAs (148). LncRNAs can be transcribed from various genomic locations in relation to protein-coding genes, including intergenic regions, intronic regions, overlapping with a specific gene on the same or opposite strand, the opposite strand of the promoter region, and enhancer regions (148, 149). While lncRNAs may have a lower degree of sequence conservation between different species, they often exhibit a high level of structural conservation (148, 150, 151). Furthermore, lncRNAs have been identified in syntenic genomic regions across species, known as locus-conserved lncRNAs, which typically serve conserved functions (150). Although lncRNAs are usually less abundant than mRNAs, they tend to display stronger tissue-specific expression patterns (152).

The sub-cellular localization of lncRNAs plays a crucial role in determining their functions (152). The majority of lncRNAs are found within the nucleus, where they associate with chromatin, while some fractions localize to the cytoplasm (147). Nuclear lncRNAs participate in various processes where they form complexes with DNA, proteins, and other RNAs (147, 152). These interactions allow them to organize the chromosomal architecture, facilitate the formation of ribonucleoprotein complexes, regulate gene transcription, and influence post-transcriptional modifications. Nuclear lncRNAs achieve these functions by mobilizing transcription factors, guiding chromatin remodeling complexes to promote histone modifications, acting as enhancers, regulating the nuclear-cytoplasmic translocation of transcription factors, and controlling the splicing of pre-mRNAs. Cytoplasmic lncRNAs, on the other hand, have distinct roles (152). They regulate the stability of mRNAs, control mRNA translation, act as scaffold molecules to stabilize ribonucleoprotein complexes, mediate protein phosphorylation, and activate signaling pathways.

#### FENDRR

lncRNAs FOXF1 Adjacent Noncoding Developmental Regulatory RNA (FENDRR) was found to be upregulated in the isolated peripheral blood mononuclear cells (PBMCs) from hypertensive patients with HFpEF as compared to healthy controls (153). FENDRR was discovered as a lncRNA essential for heart development in mice, which acts by binding the histone-modifying complexes polycomb repressive complex 2 (PRC2) and TrxG/MLL, suggesting its role as a chromatin modifier (154). FENDRR has been shown to have a protective role to the heart. It promotes the ubiquitination and degradation of p53 by increasing its binding to E3 ubiquitin ligase COP1, leading to cardiomyocyte survival in hypoxia-induced cardiomyocyte apoptosis (78) and H_2_O_2_-induced cardiomyocyte injury model (155). In contrast, it exhibited a pro-fibrotic role in a pressure overload TAC mouse model via the Fendrr/miR-106b/Smad3 pathway (156), suggesting different regulatory mechanisms of FENDRR in HFpEF vs. HFrEF.

#### CARMEN

Similar to FENDRR, Cardiac Mesoderm Enhancer-associated Noncoding RNA (CARMEN) was elevated in PBMCs of hypertensive patients with HFpEF and there was a strong positive correlation of CARMEN levels with peak VO_2_ and VE/VCO_2_ slope in HFpEF patients (153). CARMEN expression was induced during pathological remodeling in mouse and human hearts. It is essential for the differentiation of cardiac precursor cells into cardiomyocytes, and interacts with SUZ12 and EZH2, components of the chromatin-modifying complex PRC2 (79).

#### MHRT

SNPs in the lncRNA myosin heavy-chain-associated RNA transcript (MHRT) gene were associated with a risk for chronic HF (157), and was shown to be an independent predictor for HF (158). Circulating levels of MHRT were shown upregulated in hypertensive patients with HFpEF (153). In contrast, MHRT levels were downregulated in the plasma of patients with HFrEF, and patients with lower expression levels of lncRNA MHRT had worse survival compared to patients with higher expression levels (159), showing another example of the different regulatory mechanism of lncRNAs in diastolic and systolic HF. MHRT was identified as a myocardium-specific, nuclear-enriched lncRNA antisense of Myh7 with increased expression upon aging (80). Cardiac MHRT levels were decreased upon pressure overload and restoring expression of MHRT protected the animals from hypertrophy and heart failure by alleviating cardiac hypertrophy and fibrosis. MHRT executes its function by antagonizing Brg1, part of the pathological stress-activated Brg1-Hdac-Parp chromatin repressor complex in TAC mice (27-gauge needle) (80) and by promoting SUMOylation of SIRT1, leading to the activation of the PGC1-α/PPAR-α pathway in Ang-II treated neonatal rat cardiomyocytes as a model for hypertrophy (160).

#### SENCR

In a study with type-2 DM patients, serum levels of smooth muscle and endothelial cell-enriched migration/differentiation-associated long noncoding RNA (SENCR) were inversely associated with diastolic function (161). SENCR was directly associated with LV mass to LV end-diastolic volume ratio (LVMV-ratio), a marker of cardiac remodeling. SENCR was discovered as a cytoplasm-enriched lncRNA regulating smooth muscle cell contractility and migration (162). It was also found downregulated in coronary endothelial cells isolated from patients with premature coronary artery disease (CAD) (163). Reduction of SENCR levels were also found in circulating endothelial cells from early-onset coronary artery disease (EOCAD) patients, whereas an upregulation was found in circulating monocytes (164). SENCR alleviates endothelial-to-mesenchymal transition by targeting miR-126a (165) and reduces hypoxia/reoxygenation-induced cardiomyocyte apoptosis and inflammatory response by sponging miR-1 (81).

#### LIPCAR

Circulating levels of long intergenic non-coding RNA predicting cardiac remodeling (LIPCAR) was inversely correlated with diastolic function and positively associated with grade I diastolic dysfunction (161). LIPCAR was found upregulated in both HFpEF and HFrEF patients (166). Plasma levels of LIPCAR were also increased in coronary artery disease (CAD) patients with HF compared to patients with normal cardiac function (167) and were found to be a predictor for HF and cardiovascular death (168). Although the molecular mechanism of LIPCAR in HFpEF is not known, a study with atrial tissue and fibroblasts from atrial fibrillation patients, one of the most prevalent comorbidities in HFpEF, suggests that LIPCAR has a role in cardiac fibrosis via modulating the TGF-β/Smad pathway (82).

#### MIAT

Similar to SENCR, serum levels of myocardial infarction associated transcript (MIAT) were an independent predictor for increased LV mass to LV end-diastolic volume ratio (161). MIAT was first described as a risk gene for predicting AMI and a predictor for LV dysfunction with reduced EF (169, 170). *In vivo* silencing of MIAT reduced cardiac fibrosis and improved cardiac function, potentially by targeting miR-24 and therefore regulates the expression of fibrosis-related regulators Furin and TGF-β1 (83). In a STZ-induced DM mouse model, knockdown of MIAT partially restored systolic and diastolic function and alleviated cardiac fibrosis and inflammation (171). In another STZ-induced model for DM using male Sprague–Dawley rats, MIAT was found to function as a sponge for miR-22-3p and regulate the expression of death-associated protein kinase 2 (DAPK2) (172), promoting cardiac fibrosis through the PI3K/Akt signaling pathway (173). Overall, in both diastolic and systolic HF models, MIAT appears to mediate similar pathophysiological responses, namely fibrosis and inflammation.

#### CASC7

Cancer susceptibility candidate 7 (CASC7), a lncRNA frequently linked to cancer, was shown to be also associated with HF (174–176). Elevated expression levels of CASC7 were observed in both plasma samples and peripheral blood monocytes derived from HF patients, including the HFpEF patient group. Given its high diagnostic value, CASC7 was considered a promising biomarker for HF (176). Mechanistically, CASC7 was observed to be a competing endogenous RNA for miR-30c in H92C cells which subsequently inhibits IL-11 expression. It is known that elevated IL-11 expression promotes cardiac fibrosis by activating cardiac fibroblasts-mediated ECM synthesis (84, 85).

#### TUG1

Another lncRNA suggested as a promising biomarker for HFpEF is taurine upregulated 1 (TUG1). First identified as a regulator in the developing retina and brain, multiple reports currently describe TUG1 involvement in cardiovascular disease, including its possible role in HFpEF (177–182). TUG1 was increased in the serum of elderly hypertensive HFpEF patients and was confirmed to be suitable for the diagnosis of HFpEF (183), showing its diagnostic potential. TUG1 inhibition improved diastolic dysfunction in a diabetic cardiomyopathy model of db/db mice. Here, TUG1 inhibition did not interfere with diabetes-induced metabolic characteristics, implicating its direct effect on cardiac function. Knockdown of TUG1 mitigated cardiac hypertrophy and decreased cardiac fibrosis *in vivo*, and attenuated the hypertrophic response in cardiomyocytes treated with high glucose *in vitro* (86, 87). Furthermore, Chitinase-3-like protein 1 (CHI3L1) was found to promote cardiac fibrosis through upregulation of TUG1 in mice treated with Ang II (184). In summary, lncRNA TUG1 emerges as a multifaceted regulator with potential implications in both cardiac hypertrophy and fibrosis, suggesting its potential as a driver for HFpEF pathogenesis.

#### MALAT1

One of the most widely studied lncRNAs is metastasis associated lung adenocarcinoma transcript 1 (MALAT1), also referred to as noncoding nuclear-enriched abundant transcript 2 (NEAT2). MALAT1 exhibits a high degree of conservation across mammalian species and plays pivotal roles in numerous physiological processes, often implicated in the development of some cancers (185, 186). Similarly, MALAT1 displayed elevated expression in CMs exposed to high glucose conditions and in myocardial tissues from diabetic rats. Intriguingly, silencing of MALAT1 resulted in reduced CM death, improved cardiac function and morphological characteristics (89, 187). In addition, MALAT1 was able to aggravate myocardial fibrosis in hypertensive rats (188). MALAT1 also regulates the function of endothelial cells. MALAT1 knockdown in diabetic rats improved retinal endothelial cell viability and migration, leading to attenuation of retinal vessel impairment and inflammation (88). Moreover, silencing of MALAT1 was found to promote vascularization *in vivo* through a reduction in endothelial cell proliferation and an induction of pro-migratory response (189). Overall, these studies showcase the role of MALAT1 in CMs and endothelial cells in driving cardiac pathology induced by diabetes, a prevalent comorbidity in HFpEF.

#### Meg3

Another interesting lncRNA that was found to contribute to cardiac fibrosis and diastolic dysfunction is maternally expressed 3 (Meg3). Meg3 was found highly expressed in cardiac fibroblasts. Meg3 was a regulator of metalloproteinase-2 (MMP-2) production and targeting Meg3 *in vivo* effectively prevented cardiac Mmp-2 induction and decreased fibrosis, which in turn improved diastolic function by hampering cardiac remodeling (90). Apart from its role in fibrosis, Meg3 was also described to be involved in DM-induced endothelial dysfunction (91, 190). Meg3 aggravated inflammation in endothelial cells via TGF-β and Wnt/β-catenin signaling and inhibited endothelial proliferation and angiogenesis *in vitro* (92, 93). In addition, MEG3 was positively correlated to hypertension in IVF offspring, together with lowered levels of eNOS and VEGF expression, inducing endothelial dysfunction (191). Together, these reports indicate a plausible role of Meg3 in HFpEF by promoting fibrosis and endothelial dysfunction and point to its potential as a target for therapy in HFpEF.

#### HOTAIR

LncRNA HOX antisense intergenic RNA (HOTAIR) was demonstrated to decrease in diabetic mouse hearts and its knockdown in high glucose-induced H9c2 cells resulted in increased oxidative injury, inflammation and apoptosis. HOTAIR influences PTEN expression by functioning as a competitive RNA. Cardiomyocyte-specific HOTAIR overexpression in STZ-induced diabetic mouse hearts resulted in improved cardiac function along with a decrease in inflammation, oxidative stress and myocyte death (96). Moreover, HOTAIR has been implicated in endothelial dysfunction and inflammation as a consequence of diabetic complications (94, 95), further establishing its involvement in diabetes-related pathophysiology of HFpEF.

#### KCNQ1OT1

Beyond its role in cancer, the lncRNA KCNQ1 opposite strand/antisense transcript 1 (KCNQ1OT1) has acquired attention for its involvement in cardiovascular disease as it regulates cardiomyocyte apoptosis (192–196). KCNQ1OT1 expression is not only higher in serum of DM patients, but also in high glucose-stimulated primary cardiomyocytes and cardiac tissue from STZ-induced diabetic mice. KCNQ1OT1 silencing led to an amelioration of pyroptosis in cardiomyocytes and in diabetic mice through inhibition of caspase-1 via miR-214 (197). Knockdown of KCNQ1OT1 also reduced cell size and attenuate cardiac hypertrophy induced by Ang-II in cardiomyocytes via targeting miR-30e-5/ADAM9 axis (97).

#### SARRAH

SCOT1-antisense RNA regulated during aging in the heart (Sarrah) is an aging-regulated lncRNA with anti-apoptotic effects in cardiomyocytes (98). Sarrah was downregulated in cardiomyocytes of aged mice and associated with apoptosis, as inhibition of Sarrah reduced caspase activity in mouse and human cardiomyocytes. Furthermore, Sarrah was downregulated in the hearts of rats with a HFpEF phenotype. Knockdown of Sarrah in primary neonatal rat cardiomyocytes reduced contraction amplitude, contraction velocity and relaxation velocity, the latter reflecting diminished cardiac function in HFpEF phenotype. In an acute myocardial infarction mouse model, Sarrah tissue levels were downregulated in the infarcted region and overexpression of Sarrah had beneficial effects on recovery in these mice. Sarrah binds to the promotor region of its target genes, thereby forming a RNA-DNA triple helix. One of its directs targets is NRF2, regulating cell viability and ROS levels. Sarrah levels were also decreased in the right atrial appendage of atrial fibrillation patients (198). However, serum levels of Sarrah were increased, suggesting disposal of Sarrah out of cells upon injury.

#### H19

Similar to KCNQ1OT1, lncRNA H19 was also reported to be a regulator of cardiomyocyte apoptosis. A reduction of H19 expression levels was found in a rat diabetic cardiomyopathy model. Interestingly, H19 overexpression in the diabetic rats ameliorated oxidative stress, inflammation, apoptosis and fibrosis, leading to improved LV function through downregulation of apoptosis-related gene voltage-dependent anion channel 1 (VDAC1) or inhibiting ER stress (102, 197). Furthermore, H19 overexpression was shown to alleviate hypertrophic response in isoprenaline-induced hypertrophy models by regulating SMAD4 via sponging miR-145-3p (103). Moreover, H19 knockout mice showed severe HF upon pressure overload and cardiomyocyte-targeted murine and human AAV9-mediated H19 therapy was able to improve cardiac function (198). LncRNA H19 is also involved in the development of cardiac fibrosis. H19 levels are high in cardiac fibroblasts and fibrotic tissues, and its overexpression lowers dual specificity phosphatase 5 (DUSP5) levels and improves proliferation of cardiac fibroblasts (101). In addition, H19 upregulation promotes increased proliferation and synthesis of ECM-related proteins. through inhibition of the miR-29a-3p/miR-29b-3p-VEGFA/TGF-β axis (100). Moreover, H19 was identified as a negative regulator of eNOS and NO signaling in endothelial cells under hypoxic stress (99). The specific function of H19 in a specific cell type warrants precise targeting of this lncRNA in order to exploit its beneficial impact in treating HFpEF (149).

#### CRNDE

LncRNA Colorectal neoplasia differentially expressed (CRNDE) has surfaced as another player in cardiac fibrosis development. Overexpression of CRNDE was able to alleviate fibrosis and improve cardiac function in diabetic cardiomyopathy mice fed with HFD and treated with STZ. As CRNDE is relatively highly expressed in heart tissue and conserved in human, this lncRNA display a potential as is a considerable intervention target for HFpEF (199).

### Potential circRNA candidates in the pathophysiology of HFpEF

Circular RNAs (circRNAs) represent a group of single-stranded RNAs that form a covalently closed circular structure unlike traditional linear RNAs. Their lack of a polyadenylated tail renders circRNAs rather insusceptible to degradation by RNA exonucleases and thus suitable as a stable biomarker (200–203). CircRNAs are formed via back-splicing of premature messenger RNAs (pre-mRNAs). The cyclization process can occur through (1) intron-pairing-driven circularization, where flanking introns contain complementary sequences (e.g., ALU) that directly align, (2) RNA-binding protein (RBP)-driven circularization, or (3) lasso/lariat-driven circularization (204, 205). The majority of circRNAs originates from exons (ecircRNA) and are typically transported to the cytoplasm. Nonetheless, a subset of circRNAs is formed from introns (icircRNA) or both exons and introns (eicircRNA) and remains in the nucleus (206, 207).

CircRNAs are abundantly expressed in human cells and their expression often much higher than of their linear host gene, as multiple isoforms can be processed through alternative splicing (208, 209). Furthermore, they commonly exhibit cell-specific, tissue-specific and developmental stage-specific expression patterns and show differential expression profiles between physiological and pathophysiological conditions (210–213). CircRNAs are most well-known for their function as miRNA sponge or decoy, generally resulting in elevated expression levels of miRNA-targeted mRNA (214–216). CircRNAs can interact with RNA-binding proteins (RBPs), act as scaffolds or recruit proteins to specific sites, thereby enhancing processes like transcription, translation, splicing and more (207, 217). Although the bulk of circRNAs are considered non-coding, a small group of cytoplasmic circRNAs is capable of being translated (218–220), and by definition are no longer non-coding transcripts and are out of scope of this review. circRNAs have been found differentially regulated in patients with DM and diabetic cardiomyopathy (221–223). Further, circRNAs are involved in in cardiac inflammation and endothelial dysfunction (224–229). Moreover, circRNA microarray analysis on plasma samples from HF patients displays differentially regulated circRNAs like circ_0112085, circ_0062960, circ_0053919 and circ_0014010 that are significantly higher expressed in HF patients. From this dataset selection, circ_0062960 garnered attention as a compelling candidate for a potential biomarker as levels correlated with serum B-type natriuretic peptide (BNP) levels, an established clinical indicator of possible heart failure (230, 231). Below, we will further discuss several circRNAs that have been described HFpEF-related pathophysiology, including cardiac fibrosis, hypertrophy, senescence, diabetic cardiomyopathy and endothelial dysfunction.

#### circRNA_010567 and circRNA_000203

circRNA_010567 was upregulated in the myocardium of diabetic mice and cardiac fibroblasts treated with Ang II. Knockdown of circRNA_010567 was able to suppress secretion of fibrosis-associated proteins *in vitro*, like collagen I, collagen III and α-smooth muscle actin (α-SMA), by acting as an endogenous sponge of miR-141 that targets TGF-β1 and promoted myocardial fibrosis (104). In addition to circRNA_010567, circRNA_000203 was upregulated in diabetic mouse myocardium and Ang-II-treated mouse cardiac fibroblasts and its overexpression induced expression of Col1a2, Col3a1 and α-SMA *in vitro* (105). Enforced circRNA_000203 expression also resulted in an increase in cell size and ANP and β-MHC levels in mouse ventricular cardiomyocytes (NMVCs). Cardiomyocyte-specific circRNA_000203 transgenic mice presented with a further loss of cardiac function and an aggravation of hypertrophy after Ang-II treatment. circRNA_000203 is able to worsen cardiac hypertrophy via targeting miR-26b-5p and miR-140-3p, resulting in higher Gata4 levels, a known regulator of cardiac hypertrophy (108, 109).

#### circFoxo3

Circular RNA forkhead box protein O3 (circ-Foxo3) was highly expressed in the hearts of old mice and patients and was correlated with senescence markers. circFoxo3 overexpression further deteriorated cardiomyopathy induced by doxorubicin while its silencing improved cardiac function. In addition, circ-Foxo3 knockdown in mouse embryonic fibroblasts inhibited senescence, while overexpression had the opposite effect. Interaction of circFoxo3 with anti-stress proteins HIF1α and FAK, transcription factor E2F1 and anti-senescent protein ID-1 was able to block their effects, leading to cellular senescence and development of cardiac fibrosis (110, 111).

#### circHIPK3

Another circRNA involved in cardiac fibrosis and hypertrophy is circular RNA homeodomain interacting protein kinase 3 (circHIPK3). Silencing of circHIPK3 was able to decrease proliferation and migration of cardiac fibroblasts and ameliorate cardiac fibrosis both *in vitro* and *in vivo* through interaction with miR-29b-3p (112). In a diabetic cardiomyopathy mouse model, myocardial fibrosis was attenuated and cardiac function was enhanced after circHIPK3 knockdown. Through suppressing miR-29b-3p, circHIPK3 upregulated Col1a1 and Col3a1, important for the development of cardiac fibrosis (113). circHIPK3 overexpression promoted proliferation, migration, and production of fibrosis-associated proteins of CFs. circ_HIPK3 knockdown in Ang-II-stimulated CFs suppressed cell proliferation. The phenotypic transformation of CFs promoted by circ_HIPK3 was also accomplished via the miR-152-3p/TGF-β2 axis (114). These studies altogether reflect the pro-fibrotic and pro-hypertrophic traits of circ_HIPK3 and more research in the context of HFpEF could provide a new and interesting angle.

#### circNFIB

TGF-β-treated primary adult CFs displayed elevated levels of circular RNA nuclear factor I B (circNFIB). Furthermore, circNFIB overexpression inhibited CF proliferation based on TGF-β stimulation, whilst inhibition of circNFIB promoted proliferation (232). circNFIB was found to mitigate myocardial fibrosis induced by SO_2_ through suppression of the Wnt/β-catenin and p38 MAPK signaling pathways (233). circNFIB overexpression could lay a new foundation for a novel option in treating HFpEF.

### Circ_0018553

Endothelial progenitor cell-derived exosomal circ_0018553 was found to be protective for cardiac hypertrophy. Enforced expression of circ_0018553 ameliorated CM hypertrophy. Functionally, circ_0018553 sponged miR-4731 which targets sirtuin 2 (SIRT2) expression, a deacetylase that protects against cardiac hypertrophy (234, 235). The anti-hypertrophic capacity of circ_0018553 could serve as an interesting element in treating HFpEF.

#### circBPTF and circ_0071269

Circular RNA Bromodomain Finger Transcription Factor PHD (circBPTF) was found to be highly expressed in human umbilical vein endothelial cells (HUVECs) exposed to HG. miR-384 was identified as a downstream target of circBPTF (115), which subsequently targets Lin-28 Homolog B (LIN28B). Silencing of circBPTF was able to ameliorate HG-induced adverse effects, including oxidative stress and inflammation *in vitro* (236). circ_0071269 was elevated in H9c2 after HG treatment and its knockdown promoted cell viability and inhibited pyroptosis *in vitro*. Silencing of circ_0071269 was shown to attenuate cardiac dysfunction in mice with diabetic cardiomyopathy, by sponging miR-145 and thereby upregulating Gasdermin A (GSDMA) (116), an important regulator of pyroptosis (117).

#### circ_HECW2

circ_0118464, which corresponds to HECW2 gene, was highly upregulated in epicardial adipose tissue of HFpEF patients, as revealed in a genome-wide screening for circRNAs (237). Similarly, Hecw2_0009 level increased in an *in vivo* mouse study 2 or 4 weeks after TAC surgery. Using a gene set enrichment analysis, this circRNA was identified to play a role in cardiac fibrosis and hypertrophy (238). Furthermore, studies in human brain microvascular endothelial cells suggest a role for circ_HECW2 in the regulation of inflammation (239, 240), suggesting that it may also play a role in regulating inflammatory pathway in HFpEF.

## Discussion

Despite our growing understanding on the pathogenesis of HFpEF, there are still very limited effective treatment options for this disease, in particular when comparing it to HFrEF. Patients with HFpEF are heterogeneous, exhibiting different clinical phenotypes, which are associated with different pathophysiologies. One patient group may display predominantly inflammatory and cardiac microvascular dysfunction, while others are characterized more by intrinsic cardiomyocyte dysfunction and fibrosis. This heterogeneity can be determined by age onset, disease progression, where early and late HFpEF may display different phenotypes, or by different set of comorbidities that patients have. Therefore, patient stratification or phenotyping is important to identify specific treatment groups that give the best response for a specific treatment.

The lack of treatment can also be attributed to a lack of consensus on pre-clinical models used to study HFpEF and dissect its molecular mechanisms. For pre-clinical *in vivo* models, mimicking multiple comorbidities are encouraged for future studies than modeling only a single risk factor. This single hit model may still be used to interrogate the effect of individual co-morbidity that occur in patients. As for *in vitro* models, single cell model with only cardiomyocytes may not be sufficient, since cardiac microenvironment also plays a role in the development of the disease. Therefore, multi cell type model, using co-culture system or 3D heart model incorporating other cardiac cells, including endothelial cells, immune cells and fibroblast, offer a better mean to simulate the pathophysiology of human HFpEF.

As highlighted above, ncRNAs can be detected in extracellular fluid, including circulating plasma, and their expressions are altered in HFpEF, underlining their diagnostic potential. ncRNAs can enter the circulation encapsulated in extracellular vesicles or apoptotic bodies which protect them from enzymatic degradation. As compared to miRNAs and lncRNAs, circRNAs are a closed-loop structure which renders a higher resistance toward degradation by RNase. In addition, PBMCs offer another easily accessible material to assess ncRNA expression. The stability of ncRNAs in readily obtainable bodily fluids and their distinct expression patterns in HFpEF as compared to control or HFrEF render them especially intriguing as a new category of non-invasive markers for diagnosing HFpEF. miR-21 is an example of a miRNA that was found to be associated with endothelial dysfunction in HFpEF patients with DM2 and its circulating levels were downregulated after treatment with SGLT2 inhibitor. Another example of ncRNAs as a potential biomarker is LIPCAR. Its circulating levels were identified as an independent predictor for diastolic dysfunction in patients with DM2. Further, lncRNA TUG1 has been confirmed to be suitable for the diagnosis of HFpEF as it is increased in the serum of elderly hypertensive HFpEF patients. In addition, ncRNAs can be used as biomarkers to distinguish HFpEF from HFrEF (241), as described above for the combination of 7 miRNAs (miR-30b, let-7a-5p, miR-107, miR-125a-5p, miR139-5p, miR-150-5p and miR-342-3p). An overview of ncRNAs discussed in this review with potential use as biomarker for HFpEF is summarized in Table 2. As emphasized earlier, modulation of ncRNAs holds promise in improving cardiac function in HFpEF pathophysiology, underscoring their potential as novel treatment targets (Table 3). A particular ncRNA may play different role in HFpEF vs. HFrEF: it may convey a protective role in one while detrimental in the other. It may be driven by different regulation of the upstream pathways of the ncRNAs by the two different HF phenotypes, and should be considered when choosing silencing or overexpression treatment strategies. ncRNAs, lncRNAs in particular, exhibit different functions with respect to their subcellular localization. Moreover, ncRNAs can have different function in different cell or tissue types. All of these factors are crucial to consider when utilizing ncRNA-based therapies as treatment options.

**Table 2:** Use of non-coding RNAs as biomarker for HFpEF.

ncRNA	Sample	Regulated in disease	Reference
miR-21	Blood	Up	(122)
miR-29	Plasma	Down	(126–128)
miR-30b	Plasma	Down	(130, 131)
miR-30c	Plasma	Down	(132)
miR-34	Plasma	Down	(133)
miR-92	Blood	Up	(122)
miR-181	Plasma	Up	(74, 138)
miR-200c	Plasma	Up	(140)
miR-208a	Serum	Up	(141)
miR-208b	Isolated PBMCs	Up	(142)
let-7a-5p	Plasma	Down	(131)
CARMEN	Isolated PBMCs	Up	(153)
CASC7	Plasma, PBMCs	Up	(176)
FENDRR	PBMCs	Up	(153)
LIPCAR	Serum, plasma	Up	(161, 166, 167)
MHRT	PBMCs	Up	(153)
MIAT	Serum	Up	(161)
SENCR	Serum	Up	(161)
TUG1	Serum	Up	(242)

**Table 3:** Non-coding RNA-based therapeutics for the treatment of HFpEF.

ncRNA	Disease model	Regulated in disease	Treatment	Target	Reference
miR-21	db/db diabetes mouse	Down	rAAV delivery of miR-21	GSN	(124)
miR-30	db/db diabetes mouse	Up	LNA inhibitor	Fatty acid metabolism	(70)
miR-92a-2-5p	db/db diabetes mouse	Down	rAAV delivery of miR-92a-2-5p	Cytochrome-b	(72)
miR-208	Dahl salt-sensitive HF rat	Up	AntimiR-208a	Myh7	(76)
let-7b-5p	db/db diabetes mouse	Down	rAAV overexpression	Cytochrome-b, IRS1	(72)
MIAT	STZ-induced diabetes mouse	Up	Lentivirus shRNA	miR-214	(171)
TUG1	db/db diabetes mouse	Down	Lentivirus siRNA	miR-499-5p	(87)
	STZ-induced diabetes mouse	Down	Lentivirus shRNA	miR-145a/b	(86)
MALAT1	STZ-induced diabetes rat	Down	shRNA	NLRP3	(89)
	STZ-induced diabetes rat	Down	shRNA adenovirus	p38/MAPK	(88)
HOTAIR	STZ-induced diabetes mouse	Up	AAV2 overexpression	miR-34a/SIRT1	(96)
	STZ-induced diabetes mouse	Down	AAV-shRNA	LSD1	(95)
KCNQ1OT1	STZ-induced diabetes mouse	Down	Lentivirus-shRNA	miR-214-3p	(197)
H19	STZ-induced diabetes mouse	Up	Lentiviral overexpression	miR-675	(102)
	STZ-induced diabetes mouse	Up	Lentiviral overexpression	PI3K/AKT/mTOR	(197)
	ISO-induced cardiac hypertrophic mouse	Up	Lentiviral overexpression	miR-145-3p/SMAD4	(103)
circHIPK3	Ang II-induced cardiac fibrosis mouse	Down	AAV9 shRNA	miR-29b-3p	(112)
	STZ-induced diabetes mouse	Down	AAV9 shRNA	miR-29b-3p	(113)

ncRNA-based therapies can be achieved by utilizing antisense oligonucleotides (ASO), RNA interference (RNAi), or aptamers. ASOs are 17–22 nucleotide long single-stranded DNA molecules, which induce blockage of protein translation, mRNA degradation or modification of transcript splicing, through complementarity pairing. GapmeRs are a class of ASOs which consist of a DNA core flanked by two locked nucleic acids (LNA) sequences complementary to the target mRNA or ncRNA sequence. This modification introduces a higher stability, target specificity and RNase H activation resulting in enhanced silencing efficiency (243). One ASO that has been clinically approved by EMA for cardiovascular-related indication is Volanesorsen (244), which is indicated for familial dyslipidemia.

RNAi can be either be achieved with siRNAs or shRNAs. siRNAs act by mimicking the mechanism of action of endogenous miRNAs. The difference lies in the perfect complementarity of siRNAs with a given target mRNA, whereas miRNAs require only short regions of homology (−7 nucleotide long seed sequence) (245). shRNAs take advantage of the miRNA maturation pathway by being cleaved by Dicer into a double-stranded mature product followed by loading into RISC (246). Two siRNAs that have been approved by FDA and EMA are Patisiran (247) and Inclisiran (248), which is indicated for amyloidosis and hypercholesterolemia, respectively. Another form of RNAi is anti-microRNAs (antimiRs) which are basically ASOs that are designed to be fully or partially complementary to an endogenous miRNA and prevent the interaction with its target genes. AntimiRs are also known as antagomiRs when they are conjugated to cholesterol to improve intracellular delivery. anti-miR-92a is now in clinical trial to test for its efficacy to induce angiogenesis and wound healing (249). Another ASO in clinical trial is anti-miR-132-3p which is indicated for HF. Aptamers are ∼25–40 nucleotide RNA segments that specifically bind proteins or small organic molecules. Aptamers exploit the secondary structure of nucleic acids rather than the sequence complementarity for binding. Pegaptanib was the first aptamer to reach clinical approval to be used as an intravitreal injection and acts by binding to an isoform of the vascular endothelial growth factor to combat age-related neovascular macular degeneration (250). siRNAs mainly function in the cytoplasm, and therefore may be less effective against nuclear transcripts. GapmeRs on the other hand are more promising for pharmacological silencing, as they can enter the nucleus, and are therefore able to target nuclear transcripts.

In addition to silencing of ncRNAs, therapeutic overexpression can also be used as a ncRNA-based therapy. It requires the use of viral-mediated gene delivery, nanoparticles, or RNA mimics. AAV vectors are commonly used for gene therapy approaches, although it displays relatively low packaging limit and cannot be used for transcripts longer than 3–4 kb. While AAV9 has been commonly used to target cardiac muscle cells, specific targeting of other cardiac cell types are more challenging. Nevertheless, it has been shown recently that AAV9 with PAMAM-dendrimers coating can redirect the specificity more towards cardiac endothelial cells (251), underscoring the possibility to target microvascular dysfunction in HFpEF. An alternative to AAV-mediated gene delivery is local delivery of *in vitro* transcribed RNA in a manner that is similar to the recent mRNA-based vaccines. This technology is relatively new and targeting to a certain cell type is not yet possible. One would also need to locally apply the liposome-encapsulated RNA molecules, since limiting delivery to unintended organs is difficult, especially since one cannot use a tissue-specific promoter when using *in vitro*-transcribed RNA (252).

To date, no clinical trials targeting ncRNAs in HFpEF have been performed. The field of ncRNA-based therapeutics for HFpEF is still at its infancy and may be advanced by improvement of pre-clinical models with *in vitro* multi cell type models and *in vivo* multi hit models. Considering that various ncRNAs, especially miRNAs, have been explored in clinical trials for different cardiovascular conditions, it is plausible that ncRNAs could become valuable additions to the HFpEF treatment arsenal in the future.
